# Immunogenicity and safety of a quadrivalent meningococcal tetanus toxoid-conjugate vaccine (MenACYW-TT) in adults 56 years of age and older: a Phase II randomized study

**DOI:** 10.1080/21645515.2020.1733868

**Published:** 2020-04-01

**Authors:** Judith Kirstein, Miriam Pina, Judy Pan, Emilia Jordanov, Mandeep S. Dhingra

**Affiliations:** a Advanced Clinical Research, West Jordan, UT, USA; b Clinical Development, Sanofi Pasteur, Swiftwater, PA, USA

**Keywords:** MenACYW-TT, invasive meningococcal disease, older adults, MPSV4, vaccine

## Abstract

MenACYW-TT is an investigational quadrivalent meningococcal conjugate vaccine intended for the prevention of invasive meningococcal disease (IMD) caused by serogroups A, C, W, and Y in individuals aged 6 weeks and above. This Phase II, randomized, open-label, multicenter, exploratory study assessed the safety and immunogenicity of MenACYW-TT compared with a quadrivalent meningococcal polysaccharide vaccine (MPSV4) in 301 healthy adults aged ≥56 y in the US (NCT01732627). Participants were randomized 2:1 to receive MenACYW-TT or MPSV4. Serum bactericidal assays using human (hSBA) or baby rabbit (rSBA) complement were used to measure functional antibodies against meningococcal serogroups A, C, W, and Y at baseline and 30 d post-vaccination. Safety data were collected up to 30 d post-vaccination. Proportions of study participants with hSBA titers ≥1:8 against serogroups A, C, W, and Y were increased at Day 30 compared with baseline in both vaccine groups. The proportions of participants with hSBA titers ≥1:8 after MenACYW-TT vaccination were comparable to those after MPSV4 vaccination for serogroups A and C (A: 93.8% vs. 85.1%; C: 74.9% vs. 62.8%) and distinctly higher than after MPSV4 for serogroups W and Y (W: 79.5% vs. 60.6%; Y: 80.5% vs. 59.6%). Proportions of participants with rSBA titers ≥1:8 were comparable between vaccine groups for all four serogroups. The reactogenicity profiles of both vaccines were similar. Most unsolicited adverse events (AEs) were of Grade 1 or Grade 2 intensity, and no serious AEs were reported. The MenACYW-TT conjugate vaccine was well tolerated and immunogenic in adults aged ≥56 y.

## Introduction

Invasive meningococcal disease (IMD) is a rapid-onset invasive bacterial disease with potentially life-changing consequences.^1^ IMD is caused by *Neisseria meningitidis*, a Gram-negative diplococcus found exclusively in humans. It is associated with a high rate of mortality, with a case-fatality rate of approximately 10%.^2^ *N. meningitidis* has been classified into at least 12 distinct meningococcal serogroups,^3^ six of which (A, B, C, W, Y, and X) have been identified as responsible for the majority of global IMD cases.^4,5^ Despite a number of effective licensed vaccines available, *N. meningitidis* remains a leading cause of bacterial meningitis and septicemia on a global scale.^5,6^

The epidemiology of *N. meningitidis* is highly unpredictable as disease patterns vary widely over time, with substantial geographical differences in the prevalence of serogroups and disease incidence.^4,7,8^ In the US and Europe, meningococcal serogroups B, C, and Y are currently the most common cause of IMD.^8^ In Europe, following the introduction of a conjugate vaccine against C and the consequent reduction in IMD cases associated with serogroup C, serogroup B has become the most prominent cause of IMD. Serogroup Y, typically seen among older adults in the US, has been attributed to a growing proportion of IMD cases in recent years in Europe, particularly in Scandinavia.^9^ Serogroup W is reported in low numbers in the UK and the US albeit with increases observed in England and Wales since 2009;^10,11^ and is currently the predominant serogroup in the meningitis belt of sub-Saharan Africa.^9^ The incidence of IMD cases is highest in infants and children, with smaller peaks of incidence in adolescents, and older adults. However, the case-fatality rate of IMD is highest in adults aged ≥65 y.^8,12,13^

Until recently, a quadrivalent polysaccharide vaccine against serogroups A, C, W, and Y, MPSV4 (Menomune®, Sanofi Pasteur, USA) was the only quadrivalent meningococcal vaccine available in the US for use against IMD in individuals aged >55 y. Meningococcal polysaccharide vaccines have a number of limitations, including relatively short-term protection, poor immunogenicity among young children, lack of potential to induce herd immunity and immune memory, and the risk of immune hyporesponsiveness upon repeated doses. The use of conjugate vaccines is thus generally preferred over polysaccharide vaccines.^14^ The investigational quadrivalent meningococcal (serogroups A, C, Y, and W) tetanus toxoid conjugated vaccine (MenACYW-TT) is intended for the protection of all age strata, including those aged ≥56 y. This study was performed to evaluate the safety and immunogenicity of the MenACYW-TT vaccine compared with MPSV4 in adults aged ≥56 y.

## Methods

### Study design and participants

This was a Phase II, randomized, active-controlled, open-label, multicenter study performed in adults aged ≥56 y in 12 centers across the US (NCT01732627). Participants were followed for up to 30 d following vaccination. To be eligible for inclusion, participants were required to be aged ≥56 y on the day of randomization. Participants were excluded from the trial if they were participating in another clinical trial, received a vaccine in the 4 weeks preceding the trial vaccination or planned receipt of any vaccine in the 4 weeks following the trial vaccination, had previous vaccination against meningococcal disease, had a history of meningococcal infection or were at high risk for meningococcal infection during the trial. Full inclusion and exclusion criteria are included in the supplementary materials. The study was conducted between 12 November 2012 and 17 January 2013.

Using an interactive voice response system, 301 participants were randomized, with a 2:1 ratio, on Day 0 to receive a single dose of either MenACYW-TT (201 participants, batch number UD15897) or MPSV4 (100 participants, batch number UH324AC) (which was licensed for use in those aged ≥56 y at the time the study was conducted). Participants were enrolled from two age groups 56–64 y and ≥65 y at 1:1 ratio for all treatment groups. MenACYW-TT is a quadrivalent meningococcal tetanus toxoid-conjugate vaccine containing 10 µg of each serogroup (A, C, Y, and W) and approximately 55 µg of tetanus toxoid protein carrier per 0.5 mL dose. It was provided as a liquid suspension and administered intramuscularly into the deltoid. MPSV4 is a quadrivalent polysaccharide vaccine containing 50 µg of group-specific polysaccharide antigens from each of the serogroups A, C, W, and Y and 2.5–5 mg lactose stabilizer per 0.5 mL dose. It was provided as a powder and re-suspended in diluent before subcutaneous injection into the upper arm. Due to the differences in vaccine preparation and administration, this was an open-label study; the laboratory technicians were, however, blinded to the group assignment.

The study was approved by the appropriate independent ethics committees and institutional review boards prior to the start of the study. The conduct of this study was consistent with standards established by the Declaration of Helsinki and compliant with the International Conference on Harmonization guidelines for good clinical practice as well as with all local and/or national regulations and directives. Informed consent was obtained from all study participants.

### Immunogenicity

All participants were required to provide a pre-vaccination blood sample at Day 0 and a post-vaccination sample at Day 30 (up to 44 d post-vaccination). Functional antibody titers against meningococcal serogroups A, C, W, and Y were measured pre-vaccination and at Day 30 post-vaccination by serum bactericidal assays using human complement (hSBA) or baby rabbit complement (rSBA). The hSBA^15^ (Global Clinical Immunology Labs; Sanofi Pasteur) and rSBA^16^ (Public Health England) assays were conducted on sera, with serogroup-specific meningococcal bacteria and human or rabbit complement. Following incubation steps, the number of bacterial colonies was recorded and the endpoint titer determined by the reciprocal serum dilution yielding ≥50% killing as compared with the mean of the control wells. The lower limit of quantitation of both assays was a titer of 1:4. For hSBA, antibody titers ≥1:8 were considered seroprotective for each serogroup; although a titer of ≥1:4 could be considered protective, the higher titer was chosen as a conservative assumption.^17^ Subjects were considered as seroprotected when post-vaccination rSBA titers were ≥1:8 which correlates with a seroresponse against IMD.^18^

### Safety

All participants were observed for 30-min post-vaccination; with any unsolicited systemic adverse events (AEs) occurring during that time recorded as immediate unsolicited systemic AEs in the case report form. Collection of solicited AEs from Day 0 to Day 7 post-vaccination was undertaken by the individual participants with the use of a diary card. Participants were instructed to measure daily body temperature, intensity of any systemic reactions (headache, myalgia, and malaise), and any injection site reactions (pain, erythema, and swelling), and record the action taken for the event, if any. Unsolicited AEs and serious AEs (SAEs) were reported throughout the duration of the trial.

### Statistical analyses

The overall study cohort of 301 subjects provided a probability of approximately 95% of observing any AE with a true incidence of 1%. In each subgroup of 100 participants, there was a probability of approximately 95% of observing any AE with a true incidence of 3%. No imputations were performed for missing data.

This was a descriptive Phase II study to provide safety and immunogenicity data, as such no hypotheses were tested. The immunogenicity analyses were based on the per-protocol analysis set (PPAS), which included all participants who received at least one dose of the study vaccine, had at least one valid serology result, and were without major pre-defined protocol deviation (Supplementary Materials). For immunogenicity analyses, functional antibodies to the meningococcal serogroups (A, C, W, and Y) measured by hSBA and rSBA at Day 0 and Day 30 were described by the proportion of participants with titers ≥1:8, the proportion with a vaccine seroresponse (defined as a post-vaccination titer ≥1:8 if baseline titer is <1:8 or a ≥4-fold increase if baseline titer is ≥1:8), the proportion with a >4-fold increase in titers, and calculation of the geometric mean titers (GMTs). In general, the exact binomial distribution (Clopper–Pearson method) was used for calculating the confidence intervals (CI) for proportions. The 95% CIs of GMT point estimates were calculated using normal approximation, assuming they were log-normally distributed.

For safety evaluations, the safety analysis set (SafAS) included all participants who received at least one dose of study vaccine and had any safety data available. For single proportions, the 95% CIs of point estimates were calculated using the exact binomial distribution (Clopper–Pearson method).

## Results

### Participants

A total of 301 participants were enrolled and randomized 2:1 to either MenACYW-TT (n = 201) or MPSV4 (n = 100); all 301 participants provided a blood sample at Day 0. All participants but one from the MenACYW-TT group completed the study; the reported reason for early discontinuation was ‘voluntary withdrawal not due to an AE’ (Figure S1). The PPAS included 289 participants (MenACYW-TT, n = 195, MSPV4, n = 94). There were twelve participants with protocol deviations: three participants did not meet all protocol-specific inclusion/exclusion criteria (MPSV4, n = 3), the vaccine was not prepared/administered as per protocol in one instance (MPSV4), six participants either did not provide a blood sample or not within the required time window at Day 30 (MenACYW-TT, n = 5; MPSV4, n = 1), and two participants received a protocol-restricted therapy, medication, or vaccine (MenACYW-TT, n = 1; MPSV4, n = 1).

Baseline demographics were well balanced with regard to age (56.0–88.9 y) and sex, although overall there were slightly higher numbers of female than male participants in both vaccine groups (Table 1).

**Table 1.** Baseline demographics (all participants).

	MenACYW-TT		MPSV4	
	56–64 y	≥65 y	56–64 y	≥65 y
**N**	101	100	50	50
**Sex, n (%)**				
Male	45 (44.6)	35 (35.0)	20 (40.0)	25 (50.0)
Female	56 (55.4)	65 (65.0)	30 (60.0)	25 (50.0)
**Age**				
Mean (SD)	60.2 (2.5)	71.9 (5.3)	60.8 (2.6)	70.8 (5.5)
Median (min,max)	60.0 (56.0, 65.0)	70.3 (65.0, 86.8)	60.6 (56.2, 64.8)	69.2 (65.0, 88.9)
**Racial origin, n (%)**				
White	93.0 (92.1)	97 (97.0)	48 (96.0)	49 (98.0)
Asian	0 (0.0)	0 (0.0)	0 (0.0)	0 (0.0)
Black or African American	6 (5.9)	1 (1.0)	2 (4.0)	0 (0.0)
American Indian or Alaska Native	1 (1.0)	0 (0.0)	0 (0.0)	0 (0.0)
Native Hawaiian or other pacific islander	0 (0.0)	0 (0.0)	0 (0.0)	0 (0.0)
Mixed origin	1 (1.0)	2 (2.0)	0 (0.0)	1 (2.0)
**Ethnicity, n (%)**				
Hispanic or Latino	8 (7.9)	3 (3.0)	4 (8.0)	0 (0.0)
Not Hispanic or Latino	93 (92.1)	97 (97.0)	46 (92.0)	50 (100.0)

SD, standard deviation

### Immunogenicity

At baseline, the proportions of participants with hSBA titers ≥1:8 were comparable between the vaccine groups for each of the serogroups, with higher proportions of participants with titers ≥1:8 to serogroup A than the other serogroups. By Day 30 after vaccination, the proportion of study participants with hSBA titers ≥1:8 against serogroups A, C, W, and Y was markedly increased from baseline in both vaccine groups. The proportions of individuals with hSBA titers ≥1:8 at Day 30 were comparable between vaccine groups for serogroups A and C, and higher with MenACYW-TT than MPSV4 for serogroups W and Y. The proportion of participants with titers ≥1:8 was comparable across the age strata (Table 2).

**Table 2.** Proportions of participants achieving hSBA titers ≥1:8 at baseline (Day 0) and at Day 30 post-vaccination against serogroups A, C, W, and Y by vaccine group (PPAS).

		MenACYW-TT, % (95% CI)			MPSV4, % (95% CI)		
Serogroup		Total	56–64 y	≥65 y	Total	56–64 y	≥65 y
	**N**	195	98	97	94	46	48
A	Day 0	76.4 (69.8, 82.2)	79.6 (70.3, 87.1)	73.2 (63.2, 81.7)	79.8 (70.2, 87.4)	82.6 (68.6, 92.2)	77.1 (62.7, 88.0)
	Day 30	93.8 (89.5, 96.8)	95.9 (89.9, 98.9)	91.8 (84.4, 96.4)	85.1 (76.3, 91.6)	78.3 (63.6, 89.1)	91.7 (80.0, 97.7)
C	Day 0	17.4 (12.4, 23.5)	16.3 (9.6, 25.2)	18.6 (11.4, 27.7)	10.6 (5.2, 18.7)	13.0 (4.9, 26.3)	8.3 (2.3, 20.0)
	Day 30	74.9 (68.2, 80.8)	71.4 (61.4, 80.1)	78.4 (68.8, 86.1)	62.8 (52.2, 72.5)	58.7 (43.2, 73.0)	66.7 (51.6, 79.6)
W	Day 0	13.3 (8.9, 18.9)	15.3 (8.8, 24.0)	11.3 (5.8, 19.4)	8.5 (3.7, 16.1)	8.7 (2.4, 20.8)	8.3 (2.3, 20.0)
	Day 30	79.5 (73.1, 84.9)	77.6 (68.0, 85.4)	81.4 (72.3, 88.6)	60.6 (50.0, 70.6)	58.7 (43.2, 73.0)	62.5 (47.4, 76.0)
Y	Day 0	13.3 (8.9, 18.9)	12.2 (6.5, 20.4)	14.4 (8.1, 23.0)	24.5 (16.2, 34.4)	17.4 (7.8, 31.4)	31.3 (18.7, 46.3)
	Day 30	80.5 (74.2, 85.8)	81.6 (72.5, 88.7)	79.4 (70.0, 86.9)	59.6 (49.0, 69.6)	60.9 (45.4, 74.9)	58.3 (43.2, 72.4)

**Figure 1.**
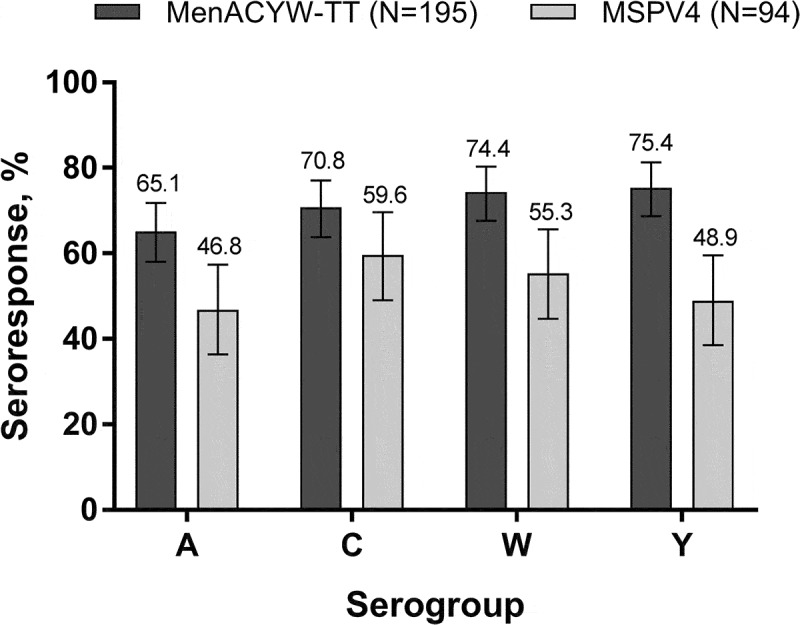
Proportion of participants with an hSBA vaccine seroresponse* at Day 30 (PPAS) *Vaccine seroresponse is defined as a post-vaccination titer ≥1:8 if baseline titer is <1:8 at baseline or a ≥4-fold increase if baseline titer is ≥1:8.

At Day 30, a larger proportion of participants had hSBA seroresponses to serogroups A, W, and Y with MenACYW-TT than with MSPV4. The proportion of participants exhibiting a seroresponse to serogroup C was comparable between vaccine groups (Figure 1). For each serogroup, comparable proportions of participants with seroresponses were seen between age strata for both vaccine groups (data not shown).

At baseline, hSBA GMTs were comparable between vaccine groups across each of the serogroups, with marked increases at Day 30 (Table 3). GMTs after MenACYW-TT were higher than those after MPSV4 for serogroups C and Y, but similar for the other two serogroups. The proportions of individuals with a ≥4-fold rise in hSBA titers after MenACYW-TT were higher than that after MPSV4 for serogroups C (65.1% vs. 44.7%), W (63.6% vs. 45.7%), and Y (70.3% vs. 42.6%), with a trend toward higher proportion observed for serogroup A (60.0% [95% CI 52.8–66.9] and 42.6% [95% CI 32.4–53.2], respectively).

**Table 3.** Geometric mean antibody titers against serogroups A, C, W, and Y at baseline (Day 0) and Day 30 post-vaccination, as assessed by hSBA and rSBA (PPAS).

		hSBA GMT (95% CI)						rSBA GMT (95% CI)					
		MenACYW-TT			MPSV4			MenACYW-TT			MPSV4		
Serogroup		≥56 y	56–64 y	≥65 y	≥56 y	56–64 y	≥65 y	≥56 y	56–64 y	≥65 y	≥56 y	56–64 y	≥65 y
	**N**	195	98	97	94	46	48	195	98	97	94	46	48
A	Day 0	9.7 (8.7, 11.0)	10.7 (8.9, 12.8)	8.8 (7.6, 10.3)	10.4 (8.6, 13.0)	10.7 (7.9, 14.5)	10.1 (8.0, 12.8)	7.8 (5.7, 10.6)	9.2 (5.7, 14.7)	6.6 (4.3, 10.0)	7.3 (4.6, 11.6)	7.1 (3.6, 14.1)	7.6 (4.0, 14.2)
	Day 30	51.0 (40.7, 63.9)	61.8 (45.1, 84.7)	42.0 (30.4, 58.0)	31.8 (22.5, 44.9)	31.5 (18.4, 54.0)	32.0 (20.2, 50.7)	1054 (825, 1346)	1496 (1095, 1971)	748 (508, 1103)	746 (501, 1109)	881 (548, 1417)	636 (334, 1212)
C	Day 0	3.3 (2.9, 3.9)	3.2 (2.7, 3.9)	3.4 (2.7, 4.3)	2.9 (2.5, 3.3)	3.2 (2.6, 4.0)	2.6 (2.2, 3.1)	3.4 (2.9, 4.2)	3.0 (2.3, 3.9)	3.8 (2.7, 5.3)	2.9 (2.3, 3.8)	2.7 (2.0, 3.6)	3.2 (2.1, 4.8)
	Day 30	48.3 (34.8, 67.1)	37.7 (24.1, 58.7)	62.2 (38.3, 101)	18.5 (12.1, 28.3)	19.5 (9.8, 38.5)	17.7 (10.3, 30.3)	1559 (1140, 2131)	1656 (1080, 2541)	1464 (920, 2331)	445 (255, 776)	692 (304, 1574)	292 (136, 624)
W	Day 0	2.8 (2.6, 3.1)	3.0 (2.6, 3.5)	2.7 (2.4, 3.1)	2.8 (2.4, 3.33)	2.8 (2.3, 3.4)	2.9 (2.2, 3.7)	4.7 (3.7, 6.1)	5.0 (3.4, 7.2)	4.6 (3.2, 6.5)	6.1 (4.1, 8.9)	3.9 (2.5, 6.1)	9.1 (4.9, 16.9)
	Day 30	29.0 (22.5, 37.3)	25.3 (17.8, 36.1)	33.2 (22.9, 48.0)	17.1 (11.6, 25.2)	14.8 (8.63, 25.5)	19.6 (11.1, 34.6)	910 (728, 1136)	941 (682, 1298)	879 (642, 1202)	493 (316, 772)	475 (242, 933)	512 (277, 946)
Y	Day 0	3.0 (2.3, 3.5)	2.9 (2.5, 3.4)	3.2 (2.5, 4.0)	3.5 (2.8, 4.4)	3.1 (2.3, 4.1)	4.0 (2.9, 5.6)	15.3 (11.4, 20.7)	17.3 (11.4, 26.3)	13.6 (8.8, 21.0)	13.1 (8.6, 20.0)	10.3 (5.6, 19.2)	16.5 (9.3, 29.3)
	Day 30	41.9 (31.8, 55.3)	36.9 (25.9, 52.5)	47.7 (31.0, 73.6)	16.6 (11.3, 24.5)	16.5 (9.6, 28.4)	16.7 (9.4, 29.7)	591 (464, 753)	569 (409, 792)	614 (428, 883)	260 (177, 382)	268 (150, 477)	252 (148, 429)

GMT, geometric mean titers; hSBA, human complement; rSBA, baby rabbit complement.

**Figure 2.**
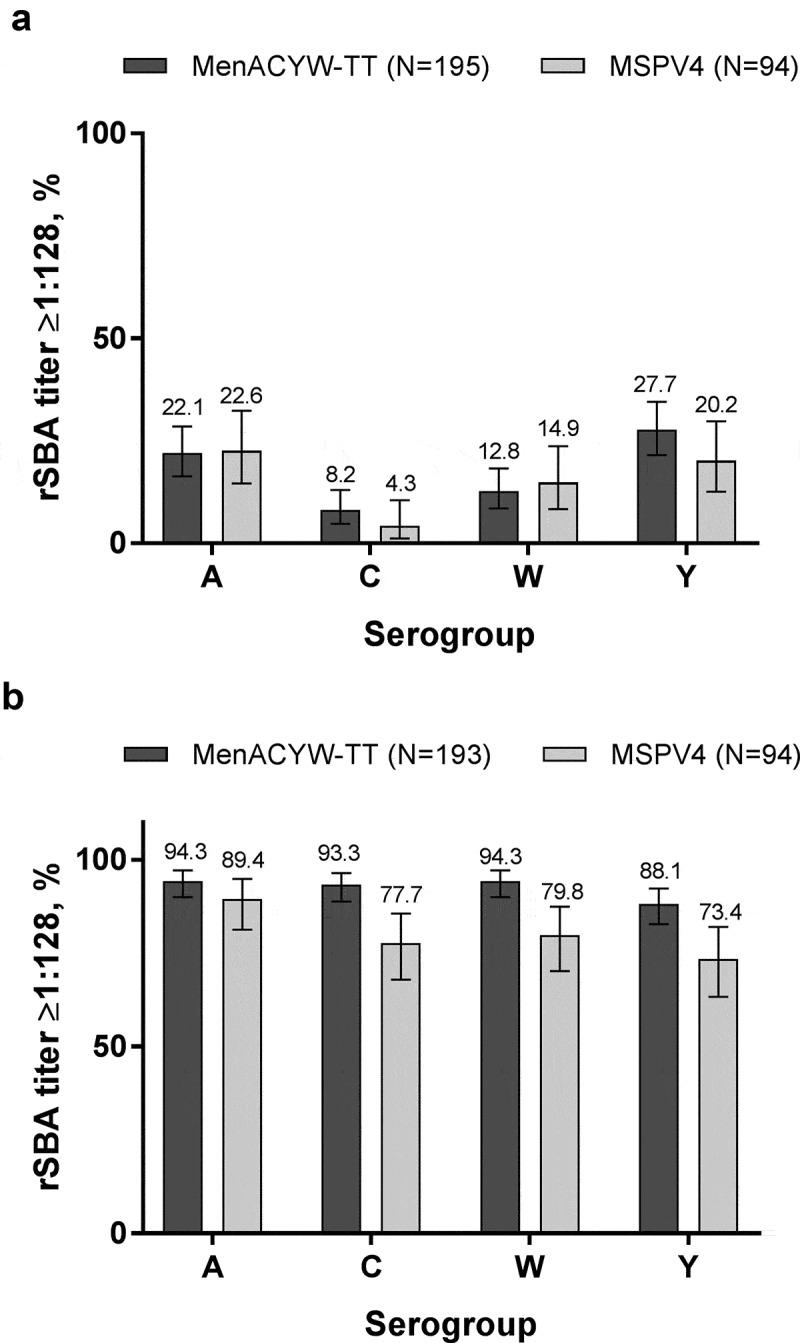
Proportion of participants with rSBA titers ≥1:128 at (a) baseline and (b) Day 30 (PPAS).

The proportions of participants with rSBA titers ≥1:128 markedly increased from baseline to Day 30 and were comparable between MenACYW-TT and MPSV4 for all serogroups (Figure 2). The results were consistent across age strata (data not shown).

At baseline, the meningococcal rSBA GMTs were comparable for all serogroups between MenACYW-TT and MPSV4 groups, with a marked increase by Day 30 (Table 3). When compared by age at Day 30, rSBA GMTs were comparable within both vaccination groups, except for serogroup C in which rSBA GMTs were higher in the MenACYW-TT group compared with the MPSV4 group in participants aged ≥65 y (Table 3). The proportions of individuals with a ≥4-fold rise in rSBA titers were comparable between vaccine groups for all serogroups and were similar between age strata in both vaccine groups (data not shown).

### Safety

Overall, the reactogenicity profile was similar for both study vaccines. A summary of safety data from Day 0 to Day 30 post-vaccination with MenACYW-TT or MPSV4 is shown in Table 4. There were no immediate hypersensitivity reactions and the proportions of participants reporting at least one solicited injection site reaction were comparable between both vaccination groups. The most commonly reported solicited injection site reaction was pain, reported by 30.7% (61/199) of participants in the MenACYW-TT group, and by 32.0% (32/100) of participants in the MPSV4 group. The proportion of participants reporting injection site erythema and injection site swelling was 11.6% and 7.6%, respectively, in the MenACYW-TT group, and 5.0% and 2.0%, respectively, in the MPSV4 group. The proportion of participants reporting at least one solicited injection site reaction was generally higher in the 56–64-year-old subset (41.4% and 42.0% in the MenACYW-TT and MPSV4 groups, respectively) than in the ≥65-year-old subset (30.0% and 28.0% in the MenACYW-TT and MPSV4 groups, respectively). The proportion of participants reporting at least one unsolicited AE was comparable between the groups. Most unsolicited AEs were of Grade 1 or Grade 2 intensity. The safety profile was generally comparable between 56–64-year-old and the ≥65-year-old subsets, apart from the small difference in the solicited injection site reactions. There were no AEs or SAEs reported that led to study discontinuation, and there were no deaths reported throughout the duration of the study.

**Table 4.** Summary of safety data from Day 0 to Day 30 post-vaccination with MenACYW-TT or MPSV4 (safety analysis set).

	MenACYW-TT (N = 199) % (95% CI)	MPSV4 (N = 100) % (95% CI)
Immediate		
Unsolicited systemic AE	0 (0.0, 1.8)	0 (0.0, 3.6)
Unsolicted AR	0 (0.0, 1.8)	0 (0.0, 3.6)
Solicited reaction	57.8 (50.6, 64.7)	53.0 (42.8, 63.1)
Solicited injection site reaction	35.7 (29.0, 42.8)	35.0 (25.7, 45.2)
Erythema	11.6 (7.5, 16.8)	5.0 (1.6, 11.3)
Swelling	7.6 (4.3, 12.2)	2.0 (0.2, 7.0)
Pain	30.7 (24.3, 37.6)	32.0 (23.0, 42.1)
Solicited systemic reaction	46.7 (39.6, 53.9)	41.0 (31.3, 51.3)
Fever	1.5 (0.3, 4.4)	1.0 (0.0, 5.5)
Headache	23.6 (17.9, 30.1)	28.0 (19.5, 37.9)
Malaise	22.1 (16.5, 28.5)	15.0 (8.6, 23.5)
Myalgia	35.2 (28.6, 42.2)	26.0 (17.7, 35.7)
Unsolicited AE	20.6 (15.2, 26.9)	17.0 (10.2, 25.8)
Unsolicited non-serious systemic AE	17.1 (12.1, 23.0)	17.0 (10.2, 25.8)
Unsolicited AR	6.5 (3.5, 10.9)	2.0 (0.2, 7.0)
SAE	0.0 (0.0, 1.8)	0.0 (0.0, 3.6)
Death	0.0 (0.0, 1.8)	0.0 (0.0, 3.8)

AE, adverse event; AR, adverse reaction; SAE, serious adverse event.

## Discussion

This Phase II, randomized, open-label study provided the first evidence of the performance of MenACYW-TT in adults aged ≥56 y. The study demonstrated that the safety of a single dose of MenACYW-TT was comparable to that of MPSV4, whilst both vaccines demonstrated good immunogenicity in vaccine naïve adults aged ≥56 years. All participants demonstrated a marked increase in both hSBA and rSBA titers 30 d after vaccination with either MenACYW-TT or MPSV4, and the differences seen between assays are consistent with previous observations of the limited correlation between assays.^19-21^ When stratified by age, immune responses were comparable within both age strata (56–64 y and ≥65 y) across vaccination groups. The overall safety profile was comparable across both vaccine groups, with no new safety concerns reported during the duration of the study.

IMD is of particular concern in older adults, with evidence suggesting higher case-fatality rates in this group compared with infants and children.^8,12,22^ A susceptibility to infection and a decline in vaccine efficacy is often seen in older individuals due to age-related changes in the immune system,^23^ intensifying the need for an efficacious vaccine with a good tolerability profile in this age strata. The only meningococcal vaccine that was licensed in the US for use in this age strata has been discontinued, resulting in the absence of a licensed meningococcal vaccine for those who are at increased risk for IMD due to travel (e.g., pilgrims to the Hajj for whom vaccination is mandatory^24^) or those who are immunocompromised. Current guidance from the Centers for Disease Control and Prevention in the US permits using a quadrivalent conjugate vaccine.^25^ Evidence from this study suggests that MenACYW-TT could be a suitable vaccine for use in this population to prevent IMD caused by serogroups A, C, W, and Y.

While this was an active-controlled trial against a vaccine licensed in the target population at the time of the study, it was a Phase II study with an open-label design due to the differences in vaccine administration, which created a risk of bias. To minimize this, laboratory technicians were blinded to vaccine assignments. No statistical hypotheses were evaluated in this study; however, the data from this exploratory study support further evaluation of the MenACYW-TT vaccine in a larger Phase III study with formal statistical hypotheses. This study also did not assess the levels of anti-tetanus antibodies following vaccination with MenACYW-TT; however, a phase II study of MenACYW-TT has shown an increase in anti-tetanus antibody levels following vaccination.^26^

MenACYW-TT was well tolerated and immunogenic when administered to adults aged ≥56 y. If the promising data from this exploratory study are confirmed in the Phase III trial, the MenACYW-TT has the potential to be a globally available alternative for the prevention of IMD in adults aged ≥56 y in countries (including the US) where there are no conjugate vaccines licensed in this age strata.

## Supplementary Material

Supplemental Material

## Article highlights

- There is no licensed meningococcal conjugate vaccine for ≥56 year-olds in the US.
- MenACYW-TT is a quadrivalent tetanus toxoid-conjugate meningococcal vaccine.
- MenACYW-TT demonstrated immunogenicity comparable to MPSV4 in older adults.
- MenACYW-TT has a similar reactogenicity profile to MPSV4.
- Conjugate vaccines have many advantages over MPS vaccines.
