# Supplementary material for: Immunogenicity and safety of a quadrivalent meningococcal tetanus toxoid-conjugate vaccine (MenACYW-TT) in adults 56 years of age and older: a Phase II randomized study
Source: Hum Vaccin Immunother. 2020 Apr 1;16(6):1299–305. doi: 10.1080/21645515.2020.1733868 (PMC7482642; doi:10.1080/21645515.2020.1733868)

Supplementary Materials

MET44 Investigators

Judith Kirstein, West Jordan, UT, USA; Derek Muse, Salt Lake City, UT, USA; Harry Geisberg, Anderson, SC, USA; Douglas Schumacher, Columbus, OH, USA; David Bolshoun, MD Denver, CO, USA; Tami Helmer, Edina, MN, USA; Randall Severance, Chandler, AZ , USA; Michael Adams, East Murray, UT, USA; Albert Tejada, Scottsdale, AZ, USA; Leslie Tharenos, St. Louis, MO, USA; James Clark, Charlottesville, VA , USA; Susan Varano, Milford, CT , USA.

Study Inclusion and Exclusion Criteria

Inclusion Criteria

1) Aged 56 to 64 years on the day of inclusion; or aged ≥65 years on the day of inclusion

2) Informed consent form has been signed and dated

3) Able to attend all scheduled visits and to comply with all trial procedures

Exclusion Criteria

1) Participant is pregnant, or lactating, or of childbearing potential (to be considered of non-childbearing potential, a female must be post-menopausal for at least 1 year, surgically sterile, or using an effective method of contraception or abstinence from at least 4 weeks prior to vaccination and until at least 4 weeks after vaccination)

2) Participation at the time of study enrollment (or in the 4 weeks preceding the trial vaccination) or planned participation during the present trial period in another clinical trial investigating a vaccine, drug, medical device, or medical procedure

3) Receipt of any vaccine in the 4 weeks preceding the trial vaccination or planned receipt of any vaccine in the 4 weeks following the trial vaccination except for influenza vaccination, which may be received at least 2 weeks before or after the study vaccines

4) Previous vaccination against meningococcal disease with either a trial vaccine or another vaccine

5) Receipt of immune globulins, blood or blood-derived products in the past 3 months

6) Known or suspected congenital or acquired immunodeficiency; or receipt of immunosuppressive therapy, such as anti-cancer chemotherapy or radiation therapy, within the preceding 6 months; or long-term systemic corticosteroid therapy (prednisone or equivalent for more than 2 consecutive weeks within the past 3 months)

7) History of meningococcal infection, confirmed either clinically, serologically, or microbiologically

8) At high risk for meningococcal infection during the trial

9) Known systemic hypersensitivity to latex or any of the vaccine components, or history of a severe reaction to the vaccines used in the trial or to a vaccine containing any of the same substances

10) Personal history of Guillain-Barré syndrome (GBS)

11) Personal history of an Arthus-like reaction after vaccination with a tetanus toxoid-containing vaccine within at least 10 years of the proposed study vaccination

12) Self-reported thrombocytopenia, contraindicating intramuscular vaccination

13) Bleeding disorder, or receipt of anticoagulants in the 3 weeks preceding inclusion, contraindicating intramuscular vaccination

14) Deprived of freedom by an administrative or court order, or in an emergency setting, or hospitalized involuntarily

15) Current alcohol abuse or drug addiction

16) Chronic illness that, in the opinion of the Investigator, is at a stage where it might interfere with trial conduct or completion

17) Moderate or severe acute illness/infection (according to Investigator judgment) on the day of vaccination or febrile illness (temperature ≥100.4°F [≥38.0°C]). A prospective subject should not be included in the study until the condition has resolved or the febrile event has subsided

18) Receipt of oral or injectable antibiotic therapy within 3 days prior to any blood draw. Should a subject receive oral or injectable antibiotic therapy within 3 days prior to any blood draw, the Investigator will postpone the blood draw until it has been 3 days since the participant last received oral or injectable antibiotic therapy. Postponement must still be within the timeframe for blood draw indicated in the Table of Study Procedures, when possible

19) Identified as an Investigator or employee of the Investigator or study center with direct involvement in the proposed study, or identified as an immediate family member (i.e., parent, spouse, natural or adopted child) of the Investigator or employee with direct involvement in the proposed study

Supplementary Figure 1. Study flow diagram

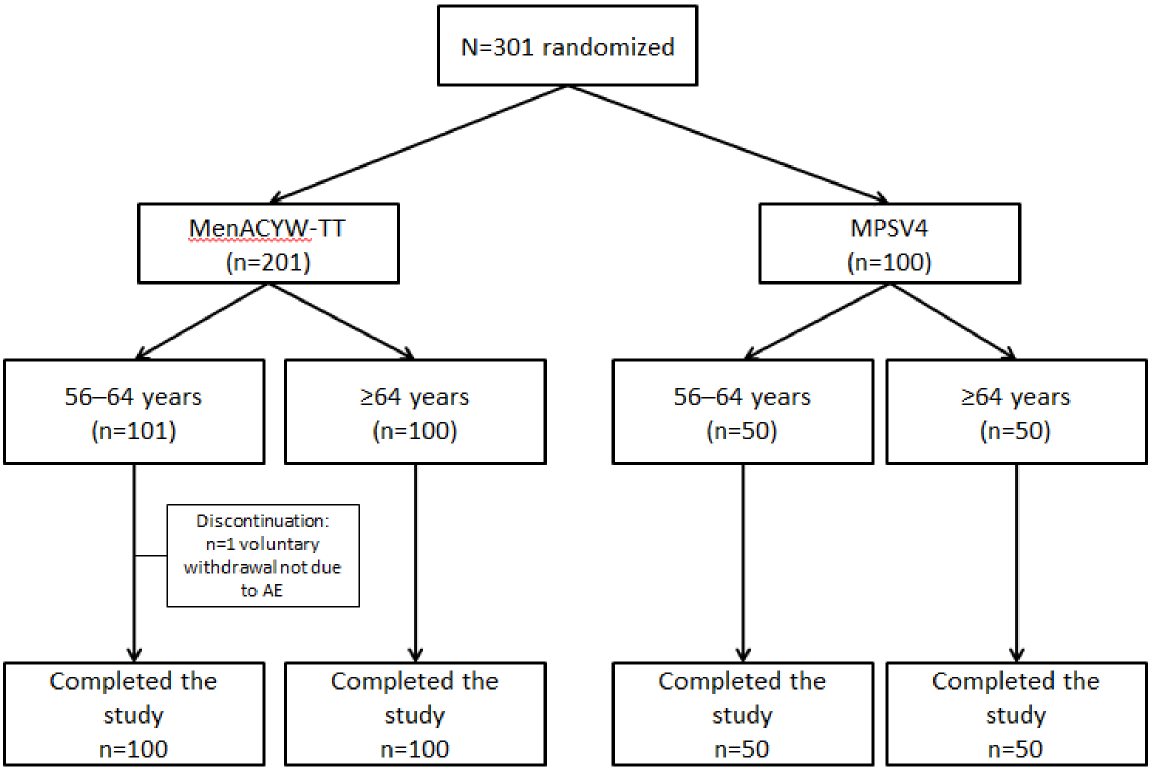

Supplement: Supplemental Material [file KHVI_A_1733868_SM3198.pdf]
